# Amyloid as a Depot for the Formulation of Long-Acting Drugs 

**DOI:** 10.1371/journal.pbio.0060017

**Published:** 2008-02-05

**Authors:** Samir K Maji, David Schubert, Catherine Rivier, Soon Lee, Jean E Rivier, Roland Riek

**Affiliations:** 1 Structural Biology Laboratory, The Salk Institute for Biological Studies, La Jolla, California, United States of America; 2 Cellular Neurobiology Laboratory, The Salk Institute for Biological Studies, La Jolla, California, United States of America; 3 The Clayton Foundation Laboratories for Peptide Biology, The Salk Institute for Biological Studies, La Jolla, California, United States of America; 4 Laboratory for Physical Chemistry, Zurich, Switzerland; Howard Hughes Medical Institute, University of California San Francisco, United States of America

## Abstract

Amyloids are highly organized protein aggregates that are associated with both neurodegenerative diseases such as Alzheimer disease and benign functions like skin pigmentation. Amyloids self-polymerize in a nucleation-dependent manner by recruiting their soluble protein/peptide counterpart and are stable against harsh physical, chemical, and biochemical conditions. These extraordinary properties make amyloids attractive for applications in nanotechnology. Here, we suggest the use of amyloids in the formulation of long-acting drugs. It is our rationale that amyloids have the properties required of a long-acting drug because they are stable depots that guarantee a controlled release of the active peptide drug from the amyloid termini. This concept is tested with a family of short- and long-acting analogs of gonadotropin-releasing hormone (GnRH), and it is shown that amyloids thereof can act as a source for the sustained release of biologically active peptides.

## Author Summary

Amyloids are highly organized protein aggregates that are associated with both neurodegenerative diseases such as Alzheimer disease and benign functions such as skin pigmentation. Amyloids self-polymerize by recruiting their soluble protein counterpart and remain stable against harsh physical, chemical, and biochemical conditions. These extraordinary properties make amyloids attractive for applications in nanotechnology. Here, we suggest the use of amyloids in the formulation of long-acting drugs, which are active over extended periods of days and weeks. Long-acting drugs have been designed to increase patient comfort, convenience, dosage accuracy, and assurance of patient compliance for drugs that have a low oral bioavailability. It is our rationale that amyloids have the properties required of a long-acting drug because they are stable depots that guarantee a controlled release of the active peptide drug from the amyloid termini. This concept is tested with a family of short- and long-acting analogs of gonadotropin-releasing hormone, and it is shown that amyloids thereof can act as a source for the sustained release of biologically active peptides.

## Abstract 3

Amyloids have the properties required of a long-acting drug because they are stable depots that guarantee a controlled release of the active peptide drug from the amyloid termini.

## Introduction

Amyloid fibrils, which are highly organized protein/peptide aggregates, are the hallmark of amyloid disease [1]. More than 20 human diseases are associated with disease-specific protein/peptide aggregation and amyloid fibril formation. For example, in Parkinson disease, the protein α-synuclein (α-Syn) and in Alzheimer disease, the amyloid peptide Aβ undergo structural alterations forming amyloid fibrils. These amyloid fibrils or conformational intermediates thereof are thought to be the toxic entity of the disease [1]. In striking contrast to the disease-associated amyloids, there are also amyloids with beneficial biological activities. For example, bacteria such as Escherichia coli express extracellular amyloid fibrils called curli that are involved in surface and cell–cell contacts promoting community behavior and host colonization [2]. The protein of chorion of the eggshell of silkworm is an amyloid that protects the oocyte and the developing embryo from a wide range of environmental hazards [3]. Yeast prions do not cause cell death, and are associated with enhanced survival of the host in certain environmental conditions [4,5]. The HET-s prion protein of the filamentous fungus Podospora anserina forms infectious amyloids that control a native function called heterokaryon incompatibility, believed to limit the spread of viral DNA [6]. Furthermore, the human protein Pmel17 forms a functional human amyloid, which appears to be important in the formation of skin pigmentation [7]. In light of these functional amyloids and other evidence, it has been suggested that the disease-associated amyloid fibrils also have a beneficial function by sequestering toxic oligomers of the amyloid protein [8].

Since proteins with nonhomologous sequences form amyloid fibrils, Dobson's group proposed that under certain conditions, many proteins and peptides can form amyloid-like fibrils [9,10], and therefore, the formation of amyloid fibrils is a generic property of the polypeptide chain [11]. Therefore it is likely that amyloid fibrils share common properties, and indeed, electron microscopy (EM) studies suggest that they all form long filaments of several hundred nanometers in length and only a few nanometers in diameter [12]. The filaments are composed of several protofilaments, and within a protofilament, hundreds of protein/peptide molecules are aligned with a cross-β-sheet topology [12,13], which enables them to bind Congo red (CR) [14] and Thioflavin T (Thio T) [15]. This structural arrangement makes amyloids inert against harsh treatment such as heat, pH, proteases, and physical forces. Furthermore, amyloids grow in a nucleation-dependent manner [16]. The properties outlined above make amyloids an attractive tool for nanotechnology and bioengineering. For example, amyloids may be used to make nanowires [17,18], as scaffolds for bioactive materials [19,20], and as a substrate for neurite outgrowth and synapse formation, as well as for tissue repair and tissue engineering [21]. Furthermore, the mechanical properties of insulin amyloids are comparable with steel [22], indicating the super stability of amyloid organization. Here, we explore the use of amyloids in a pharmaceutical application for the formulation of long-acting drugs. One possibility to make drugs long acting is their storage in a depot from which the drug is released slowly in a controlled fashion [23]. It is our rationale that an amyloid-forming protein/peptide drug has the necessary properties of a long-acting drug: (1) an amyloid is a stable reservoir containing only the peptide drug; (2) the high structural organization of the amyloid aggregate guarantees a controlled release of the peptide drug from the fibril termini; and (3) the peptide drug is active upon release. This concept for drug delivery is in direct contrast to another approach in which the amyloid propensity of a peptide is reduced for better solubility and efficacy [24].

We examined the concept of using amyloids for the formulation of long-acting drugs to a family of analogs of gonadotropin-releasing hormone (GnRH; *p*-Glu-His-Trp-Ser-Tyr-Gly-Leu-Arg-Pro-Gly-NH_2_). GnRH is produced in the hypothalamus, stimulates the secretion of gonadotropins, and thereby plays a major role in the modulation of reproductive functions [25,26]. GnRH analogs are now recognized as potential drugs for the management of sex steroid-dependent pathophysiologies, such as hormone-responsive prostate cancers, management or treatment of breast and gynecological cancers, endometriosis, precocious puberty, uterine myoma, ovarian hyperandrogenism, premenstrual syndrome, and the induction of ovulation [26–28]. Most of these disorders can be treated with long-term applications of GnRH analogs. However, because GnRH analogs are peptides, they must be administered subcutaneously on a daily basis, frequently over long periods of time. Therefore, long-acting GnRH analogs were developed with a duration of action of up to 50 d compared to a few hours for normal GnRH [29,30]. Some of these long-acting GnRH analogs exhibit concentration-dependent aggregation and form liquid crystals or gels in aqueous solution [31,32]. Powell et al. [31] observed that the addition of electrolyte affects the liquid crystal stability, as well as the temperature at which birefringent liquid crystals formed in aqueous formulations of the GnRH analog deterelix. The GnRH analog leuprolide forms β-sheet–rich aggregates and gels with increasing peptide concentration or the addition of salts [33], whereas the GnRH antagonist A-75998 exhibits aggregation and gelation in aqueous solution [32]. Furthermore, Jiang et al. [34] observed that subcutaneous injection of a solution containing the GnRH antagonist orntide forms gel at the injection site at high doses. Here, we show that gels of long-acting GnRH analogs are composed of amyloid fibrils and suggest that the duration of action of GnRH analogs depends on the ability of the amyloids to slowly release active peptide.

## Results

To address the concept that amyloid fibrils can be used for the formulation of long-acting drugs, we studied the amyloid formation and some in vivo properties of 23 GnRH analogs, wild-type GnRH, 11 short-acting analogs, and 11 long-acting analogs (listed in Tables S1 and S2)**.** The two lists of GnRH analogs provide information about their amino acid composition, the half-maximal inhibitory concentration (IC_50_) for the receptors, and their duration of action. High-affinity antagonists have been selected to cover both short- and long-acting variants, as well as a variety of amino acid sequence compositions. All analogs were assayed for their capacity to form amyloids by time-resolved Thio T binding, CR binding, and EM.

### Thio T Binding Differentiates Duration of Action by GnRH Analogs

**Figure 1:**
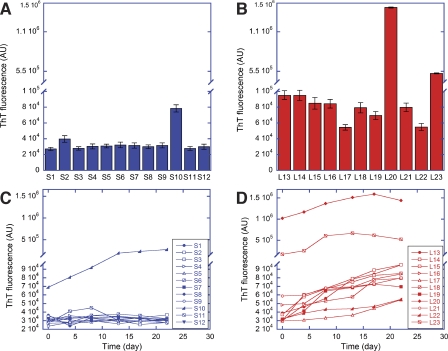
Thio T Binding of GnRH Analogs Indicative of Amyloid Fibril Formation Thio T fluorescence of (A) short-acting and (B) long-acting analogs listed in Tables 1, S1, and S2 after 21 d of incubation at room temperature without stirring. Kinetics of fibril formation of (C) short-acting and (D) long-acting GnRH analogs. The intensity of Thio T fluorescence is shown as arbitrary units (AU). The error bars represent the standard deviation of three independent experiments.

The kinetics of fibril formation in vitro is generally a slow process over several days or weeks, and can be monitored by the increase of fluorescence intensity of the cross-β-sheet–sensitive dye, Thio T [15]. Thio T fluorescence is thereby insignificant during the initial incubation period of amyloid aggregation due to the nonexistence of Thio T–sensitive β-sheet aggregates. After the initial phase, a time-dependent increase of fluorescence intensity is observed until a maximum intensity is reached, at which time maximal fibril formation has occurred. Hence, the Thio T binding study provides information about the relative rate of the formation of amyloid fibrils. For the Thio T binding studies, all GnRH analogs were incubated at room temperature at a concentration of 1 mg/ml in 5% D-mannitol, conditions identical to the formulation used in the animal experiments [29]. In addition, 0.01% sodium azide was added. The Thio T fluorescence was monitored daily for all analogs over a period of 21 d (Figure 1). With the exception of analog S10, there is no significant Thio T binding by short-acting analogs during the entire incubation period. In contrast to short-acting analogs, all long-acting analogs show a significant increase of Thio T intensity indicative of fibril formation. The long-acting analogs L20 and L23 bind significantly more Thio T during the entire experiment, which suggests that these analogs form amyloids immediately upon sample preparation. The Thio T binding studies are summarized in Figure 1A and 1B: With the exception of S10, all long-acting analogs produce three to four times more Thio T signal relative to short-acting analogs after 21 d of incubation, indicating that duration of action is correlated with amyloid formation.

**Table 1:**
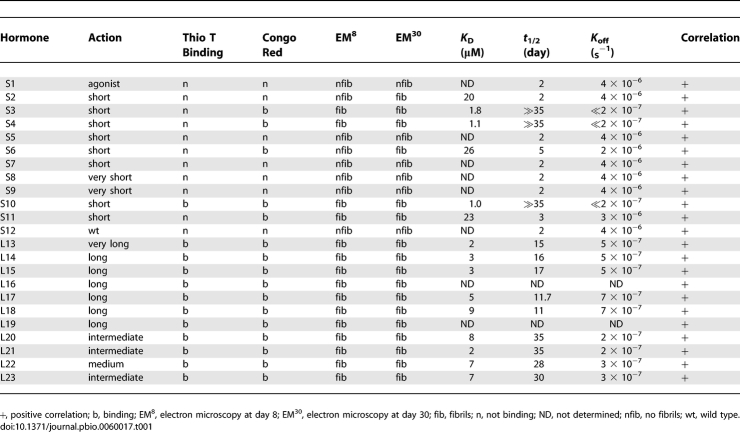
Biochemical and Biophysical Properties of Aggregates By GnRH-Analogs

### CR Binding Supports Amyloid Formation of Long-Acting GnRH Analogs

To confirm amyloid formation by GnRH analogs, CR binding was measured. CR, like Thio T, is a dye routinely used to detect β-sheet–rich assemblies [35]. CR binding can be measured by the “red shift” change of its absorption maximum from 490 to 540 nm, by an increase of the dye's molar absorptivity, and in favorable cases by eye. All three types of measurements were performed with GnRH analogs incubated for 30 d. The data (Figure S1 and Table 1) show that among 12 of the short-acting analogs, seven do not bind CR, indicative of no amyloid formation. However, five short-acting GnRH analogs, i.e., S3, S4, S6, S10, and S11, bind CR, showing that some short-acting GnRH-analogs are able to form amyloids after prolonged incubation (see also below). In contrast to short-acting analogs, all long-acting analogs bind CR.

### Electron Microscopy Supports the Formation of Amyloid Fibrils of Long-Acting GnRH Analogs

**Figure 2:**
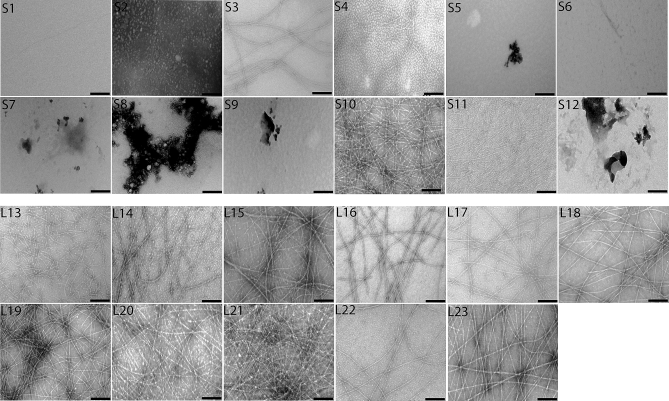
Morphologies of GnRH Analogs Incubated for 8 d Transmission electron microscopy (TEM) of negative-stained samples was performed. “S” indicates short-acting and “L” indicates long-acting GnRH analogs as labeled in Tables 1, S1, and S2. Scale bars indicate 200 nm.

To formally determine the formation of amyloid fibrils by GnRH analogs and to characterize other possible assemblies present in the sample, EM was done on all of the GnRH analogs. Since the EM analysis is for a qualitative interpretation only, amyloid fibrils and other small aggregates might be observed under the electron microscope even if only a minority of the sample forms fibrils. Samples were studied after 8 and 30 d of incubation. The EM study at day 8 (Figure 2) shows that among 12 short-acting GnRH analogs, only four (S3, S4, S10, and S11) form amyloid-like fibrils. After day 30, the EM data suggest that the short-acting analogs S1, S5, S7, S8, S9, and S12 do not form any detectable amyloid-like fibrils, but that all other short-acting analogs (i.e., S2, S3, S4, S6, S10, and S11) form amyloid-like fibrils (Figure S2). In contrast, all long-acting GnRH-analogs form amyloid-like fibrils after 8 and 30 d of incubation (Figures 2 and S2). The fibrils show a wide variety of morphologies, comprising helical ultrastructures with varying degrees of lateral association. Furthermore, the fibrils are composed of one or more filaments, resulting in overall diameters ranging from approximately 6 to 12 nm. No oligomers are observed for the long-acting compounds at day 8 or at day 30 (Figures 2 and S2). Short worm-like protofibrils are observed only for L16 at day 8 (Figure 2).

The combination of the Thio T, CR binding, and EM studies summarized in Table 1 show that all the long-acting GnRH analogs form amyloid fibrils within a short time period. Half of the short-acting analogs do not form fibrils under any of the conditions tested. However, the other half of the short-acting analogs is able to form fibrils after extended incubation times and S10 forms fibrils immediately. Therefore, amyloid fibrils of GnRH analogs appear to be required but not sufficient for a long-duration of action.

### Peptide Release by Amyloid Fibrils of GnRH Analogs

**Figure 3:**
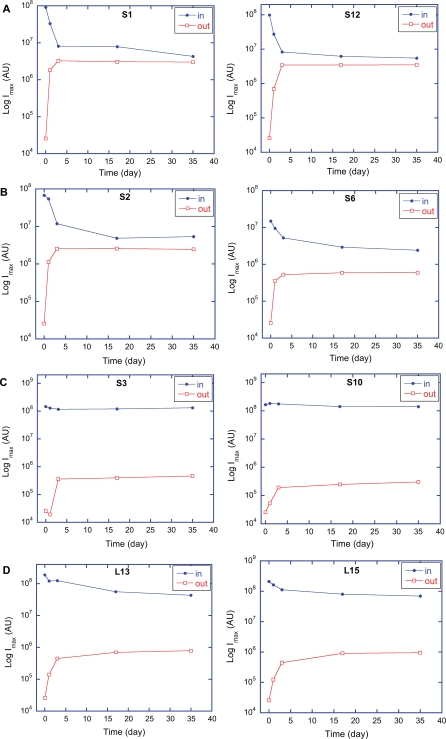
Monomer Release Profiles of GnRH Analogs from Aggregates Time-dependent monomer release was measured by the intrinsic fluorescence of the GnRH analogs with excitation at 280 nm and emission at the range of 290–500 nm. The volume- and concentration-corrected fluorescence maxima (*I*_max_) from both inside (in) and outside (out) of the membrane medium were plotted against time of incubation. The fluorescence intensity is expressed in arbitrary units (AU). A representative set of profiles are shown: in (A), the monomer release of the short-acting analogs S1 and S12, which do not form fibrils; in (B), the monomer release of short-acting analogs S2 and S6, which form unstable fibrils; in (C), the monomer release of short-acting analogs S3 and S10, which form very stable fibrils; and in (D), the monomer release of the long-acting analogs L13 and L15.

Although amyloid fibrils are very stable, they must follow thermodynamic equilibrium conditions between fibril and monomer. The fibril–monomer equilibrium is of particular importance for the proposed pharmaceutical application, because fibrils must be both stable enough to guarantee a long duration of action, and the release of active peptide must be sufficient to generate effective drug concentrations. To address these two opposing tasks, peptide release for all GnRH analogs was measured by a dialysis experiment similar to a study of the GnRH agonist leuprolide [36]. A total of 200 μl of 30-d-old GnRH analog (in) was dialyzed 4 ml of 5% D-mannitol (out). A 3.5-kDa cutoff, which is twice the size of the peptides studied, was chosen to ensure that the released particles are most likely monomeric. The peptide release and the remaining aggregates were monitored by fluorescence over 35 d at room temperature. All but two analogs (i.e., L16 and L19) produced good aggregation-independent fluorescence signal in the emission range of 290–500 nm (excited at 280 nm) and could be analyzed (see Material and Methods for more details). Figure 3 shows representative examples of the dialysis experiment displaying normalized fluorescence intensities at wavelength maxima (λ_max_) both inside (in) and outside (out) of the membrane. In Figure 3A the monomer release profile of wild-type GnRH (S12) and the short-acting analog S1, neither of which form fibrils after 30 d of incubation (Figures 1 and 2), are shown. The fluorescence intensities inside and outside of the membrane decrease and increase, respectively, within a very short period of time and reach equilibrium within 3 d (Figures 3A and S3). The *t*_1/2_ and *K*_off_ of S1 and S12 are approximately 2 d and approximately 4 × 10^−6^ S^−1^ (Table 1), representing the intrinsic diffusion of monomeric GnRH analogs through the membrane. Similar release profiles are observed for all of the short-acting analogs, which do not form fibrils after 30 d of incubation, as well as the fibril-forming but short-acting analogs S2 and S6 (Figure 3B) and S11 (Table 1). The latter data suggest that S2, S6, and S11 form unstable fibrils. In contrast, the release profiles of fibril-forming, but short-acting, analogs S3 and S10 (Figure 3C) and S4 (unpublished data) show very little fluorescence intensity changes both inside and outside of the membrane at 35 d. Therefore, the *t*_1/2_ and *K*_off_ of S3, S4, and S10 are ≫35 d and ≪2 × 10^−7^ (Table 1), respectively. The data suggest that S3, S4, and S10 form very stable amyloids with very little peptide release. This hypothesis is further supported by the finding that S4 is present as amyloid at the injection site over a period much longer than its duration of action (see below and Figure S6). It can be concluded that the release profile of all short-acting analogs indicate that short-acting analogs are of three categories: (1) not forming fibrils, (2) forming very unstable fibrils that readily dissociate into monomers, and (3) forming very stable fibrils such as S4 which release peptides so slowly that an effective concentration of the drug for action eventually is not reached.

In striking contrast, for all the long-acting GnRH analogs, a gradual decrease in fluorescence intensity inside and a corresponding increase outside of the membrane is observed between days 3 and 35 after the initial intrinsic diffusion of residual monomers within the first 3 d (Figure 3D; the residual monomer comprises thereby only 1%–5% of peptide as estimated from the relative fluorescence intensities). The half-life (*t*_1/2_) of all long-acting analogs ranges from day 15 to 30 d (Table 1). The *K*_off_ of the long-acting analogs (2 × 10^−7^ to 7 × 10^−7^; Table 1) suggest that long-acting analogs release monomer approximately ten times faster than short-acting, very stable, fibril-forming GnRH analogs such as S4.

### The Effect of Low Molecular Weight Glycosaminoglycans on Peptide Release by Amyloid Fibrils of GnRH Analogs

Monomer release in vitro may be altered in vivo by amyloid binding components from the host, such as amyloid P and glycosaminoglycans, which may stabilize amyloid fibrils [37,38]. To test the effect of glycosaminoglycans on fibril formation and peptide release, the fibril formation and monomer release profiles of samples of compounds L13 and L14 were measured in absence and presence of low molecular weight (LMW) heparin (600 μM peptide in 5% D-mannitol, 0.01% sodium azide with or without equimolar LMW heparin). After overnight incubation in the absence of LMW heparin, both L13 and L14 samples bind Thio T indicative of amyloid formation. These data are in line with earlier experiments (Figures 1 and 2). Interestingly, the presence of LMW heparin results in much higher Thio T binding for both L13 and L14 peptides (Figure S4). These findings suggest that LMW heparin induces fibril formation of peptide analogs L13 and L14. Furthermore, the dialysis experiment shown in Figure S5 suggests that the presence of LMW heparin results in the slower release of monomers and a significantly slower decay of the fibrillar material relative to the control. Therefore, host factors may influence the in vivo properties of amyloid fibrils of the GnRH peptide hormone analogs. In particular, they may stabilize the fibrils.

### GnRH Analogs Form Amyloids In Vivo

**Figure 4:**
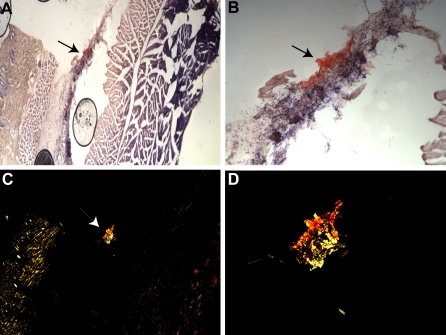
In Vivo Amyloid Formation of the GnRH Analog L14 A total of 200 μg of L14 in 200 μl of 5% D-mannitol was injected subcutaneously into an adult rat, which was sacrificed 1 d later. Tissue sections close to the injection site were stained with CR. In (A) and (B), the red CR staining, and in (C) and (D), the CR birefringence is observed indicative of in vivo amyloid formation. The pictures of (A) and (B) represent the bright-field microscope images with 5× and 10× resolutions, respectively. The same section is shown in (C) and (D) under cross-polarized light with 5× and 10× magnification, respectively.

To determine whether GnRH analogs form and/or stay as amyloids in vivo at the injection site, we injected them into adult rats and stained the potential amyloid with CR [39]. A fresh preparation of the long-acting analog L14 was subcutaneously injected at a concentration of 200 μg in 200 μl of 5% D-mannitol. The sample before injection was in a non-amyloid state as measured by EM and Thio T binding (unpublished data). Also 5% D-mannitol was injected as a control into the same animal. Twenty-four hours after the subcutaneous injection, the tissues close to the injection sites were taken for cryo sections, and the tissue sections were stained with CR. Figure 4 shows that a portion of the tissue is stained by CR and produces strong birefringence indicating the presence of amyloid fibrils. No CR binding was observed with either the control (5% D-mannitol only; unpublished data) or upon subcutaneous injection of a fresh preparation of the non-fibrillar short-acting analog S2 (unpublished data). In contrast, CR binding was observed upon injection of an aged fibril-containing S2 sample, as well as aged amyloid-containing or freshly prepared S4 samples. The presence of amyloids of the S4 sample was observed after 24 h and 7 d of subcutaneous administration, respectively (Figure S6).

These data show that GnRH analogs are able to form amyloids in vivo. The capacity of peptide drugs to form amyloids in vitro has been previously documented for insulin [40,41] .

### The Subcutaneous Administration of Amyloid Fibrils Prolongs the Duration of Action of the GnRH Analog S2

**Figure 5:**
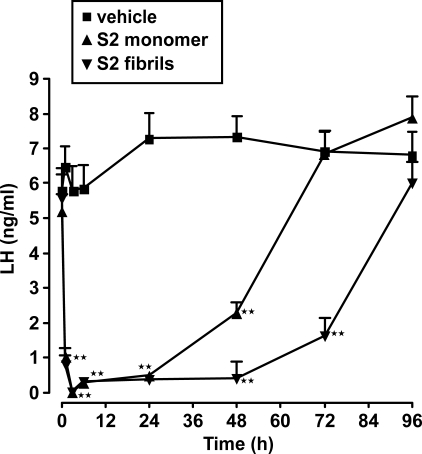
The Formation of Fibrils Inhibits LH Release in Castrated Rats Total dose was 50 μg in 50 μl (5% mannitol). Blood samples were collected at the times shown on the abscissa. Results are illustrated as mean plasma LH levels (*n* = 6 rats) ± standard error of the mean (SEM). Double asterisks (**) indicate *p* < 0.01 versus vehicle.

The correlation between fibril formation and a long duration of action suggests that a peptide administered in a fibril form might be longer-acting than in its soluble form if the latter does not form fibrils immediately upon injection. A good candidate to test this hypothesis is S2, since it is a short-acting analog that does not form fibrils upon injection (see above), but is able to form fibrils in vitro upon long incubation (Figure S2). Therefore, the duration of action of a fresh-prepared S2 and an aged fibrillar S2 sample was measured in vivo with the castrated male rat assay [29]. In short, 10 d after castration, rats (six per group) were injected subcutaneously with 50 μg of freshly prepared S2 and aged fibrillar S2 in 50 μl of 5% mannitol in parallel with a control (vehicle only). Blood sampling was performed predose, then 1, 6, 24, 48, 72, and 96 h after subcutaneous administration. The effects of the control and the two S2 samples on the gonadotropic axis were determined by measurement of plasma luteinizing hormone (LH) levels by radioimmunoassay (Figure 5) [29]. Both samples suppress LH levels in the first 24 h as expected [42,43]. However, after 72 h, the freshly prepared S2 sample lost its action, whereas the aged fibrillar S2 sample is still highly active in suppression of LH. The loss of action after 96 h of the fibrillar S2 sample is attributed to the low stability of the S2 fibrils determined in vitro (Table 1). Since the only difference between the two S2 samples is the aggregation state (soluble versus fibrillar), the difference in the duration of action is therefore due to their different aggregation state. In conclusion, the formation of fibrils of a peptide drug can prolong its duration of action.

### Toxicity of Amyloids of GnRH Analogs

**Figure 6:**
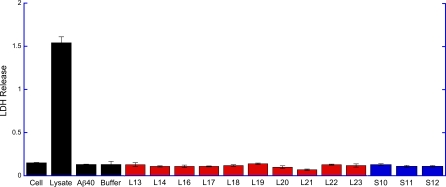
Effect on LDH Release by Hormone Fibrils The mouse embryo fibroblast cells were treated with fibrils of GnRH-analogs, buffer alone (Buffer), and Aβ(1–40) (Aβ40), and the release of LDH was assayed. The amount of LDH release is shown as arbitrary units (AU). The cell lysate and Aβ(1–40) were used as positive control, whereas cells treated with buffer alone were used as negative control (black bars). Three independent experiments were performed. “S” indicates short-acting (blue bars) and “L” indicates long-acting (red bars) GnRH analogs as labeled in Tables 1, S1, and S2.

Since amyloid fibrils can be toxic to cells and are associated with several neurological diseases [1,44], it is a concern whether or not amyloid fibrils of GnRH analogs are able to generate a toxic response as well. To measure the potential toxicity of GnRH analogs, we used the lactate dehydrogenase (LDH) release assay for cell killing [44]. The LDH release assay is a widely used assay to determine the cytotoxicity of chemicals or environmental toxic factors. LDH is a soluble cytosolic enzyme that is released into the culture medium following the loss of membrane integrity. For the LDH assay, we used mouse embryo fibroblast cells and aged peptide samples at 60 μM in 5% D-mannitol. Figure 6 shows that in contrast to the lysate as positive control, neither the peptide analog samples nor Aβ(1–40) generated toxicity in a dermal cell line. These toxicity studies suggest that amyloid fibrils of GnRH analogs are not toxic to dermal cells.

### Seeding of Wild-Type GnRH by Amyloid Fibrils of GnRH Analogs

Another concern for the application of amyloids in the formulation of long-acting drugs is the potential multiplication and spread of amyloids through seeding. Only a small amount of fibrillar seed is needed to induce and accelerate self-polymerization in the presence of monomeric amyloid protein/peptide [16]. Within the context of GnRH, perhaps amyloids of the injected GnRH analogs may act as the seeds and the host GnRH as the reservoir of the free monomer. To mimic this situation in vitro, a seeding reaction was set up with a small amount of matured, long-acting L20 fibrils (5% v/v) in presence of a solution of wild-type GnRH (95% v/v). The fibril growth of the mixture as well as the wild-type GnRH alone (S12) and mature L20 fibrils was followed over a period of 1 mo by Thio T binding (Figure S7A). There is no significant Thio T binding during the entire seeding experiment, which suggests that L20 fibrils are unable to seed wild-type GnRH. Further confirmation was obtained by EM at day 0 and after 1 mo of incubation (Figure S7B). No detectable amyloid fibrils in the mixture or wild-type GnRH alone were observed, further supporting the notion that L20 is unable to seed wild-type GnRH.

**Figure 7:**
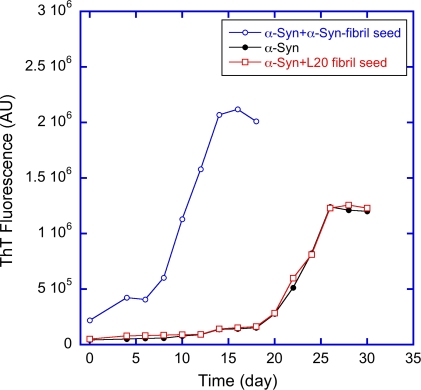
Lack of Seeding Capacity of L20 Fibrils on α-Syn The freshly prepared α-Syn solution in PBS (pH 7.4) was incubated with 5% (v/v) seed of matured L20 amyloid in 5% D-mannitol at 37 °C with vigorous stirring. α-Syn in PBS (pH 7.4) with 5% (v/v) of 5% D-mannitol was also incubated as a control. The formation of amyloid was monitored for the mixture (α-Syn+L20; red line), α-Syn in the presence (positive control; blue line) and the absence (negative control; black line) of α-Syn seed (5%) by Thio T binding. The intensity of Thio T fluorescence is shown as arbitrary units (AU) versus time.

Although the seeding of amyloids requires a high amino acid sequence similarity between the protein/peptide of the seed and its soluble host counterpart [1,45], cross-seeding between different protein species have been documented [46]. To test whether amyloids of long-acting GnRH analogs are able to cross-seed with a protein associated with Parkinson disease, α-Syn, a seeding reaction was done with a small amount of matured L20 fibrils (5% v/v) in the presence of α-Syn (95% v/v; 400 μM concentration of monomeric solution [pH 7.4] in PBS). The fibril growth of the mixture as well as α-Syn in PBS as control were followed over a period of 30 d by Thio T binding. As shown in Figure 7, α-Syn aggregates spontaneously as reported [47]. The addition of a seed of L20 fibrils did not significantly influence the aggregation of α-Syn, which suggests that the cross-seeding capacity of L20 amyloids with α-Syn is low. In contrast to the L20 fibril seeding experiment, the addition of α-Syn fibril seeds accelerated α-Syn aggregation significantly (Figure 7).

## Discussion

Drugs of peptide or protein origin usually have low oral or transdermal bioavailabilities and short in vivo half-life, and therefore require delivery by infusion, frequent injections, or subcutaneous administrations. To overcome these delivery problems, novel formulations of the drugs have been designed that release continuously over an extended period to maintain an active drug concentration within the therapeutic window. Methods for controlled drug release include implantable devices such as infusion pumps, chips, and diffusion chambers, as well as biodegradable and easy to administer hydrogels, liposomes, microspheres, and polymer depots loaded with the drug of interest [23,48,49]. Another compelling approach proposed is the crystallization of the drug followed by the administration of drug crystals, which serve as a protected reservoir that releases the active compound relatively slowly [50,51]. Such depot designs may offer numerous advantages. These include protection of the drug from enzymatic degradation, the ability to deliver the drug locally to a particular site in the body, as well as increased patient comfort, convenience, dosage accuracy, and assurance of patient compliance [52,53]. However, improved technologies and new approaches are still needed that can deliver otherwise insoluble, unstable, or unavailable therapeutic compounds to reduce the amount of drugs used, to release the drug in a “smart” manner, and to maximize the drug load, which is the amount of drug per depot [54].

**Figure 8:**
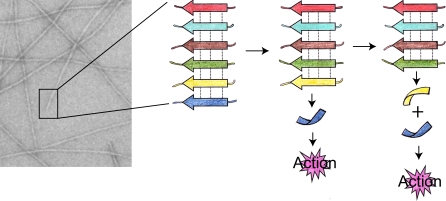
Schematic Representation of the Drug Action by Amyloids of GnRH Analogs The stable amyloid fibrils formed prior or shortly after subcutaneous injection and gradually release monomeric functional analogs at their termini. The EM picture shows amyloid fibrils of a long-acting GnRH analog. The end of a fibril is highlighted, and its structural composition of aligned peptides is schematically indicated. Each peptide colored individually in the fibril schematic interacts with neighboring peptides through intermolecular hydrogen bonds forming an “infinite” β-sheet along the fibril axis. This structural arrangement requires that peptides can be released only at the termini of the fibrils, which guarantees a controlled and slow release.

Here, we suggest the use of a peptide drug with the ability to form amyloids before or immediately after administration. This approach may simultaneously overcome two problems since the peptide drug itself forms a stable structurally organized depot, that releases monomeric active peptide slowly from the amyloid fibril termini (Figure 8). Hence, the long-acting amyloid drug consists of only one type of molecule and concomitantly the depot has a drug load of 100%. Another advantage may be that the amyloid fibril formulation may protect the biological, physical, and chemical integrity of the drug molecules in the very stable cross-β-sheet structure during processing, storage, and even upon delivery. Furthermore, amyloid binding components from the host organism such as amyloid P and glycosaminoglycans may stabilize the drug fibrils and protect them from degradation [37,38,55,56], and may reduce the potential immune response of the drug by covering the drug fibrils. In addition, an amyloid drug may be easily administered by subcutaneous injection, it is highly concentrated since it is already an aggregate, and the amyloid depot can easily be located close to the injection site and removed if necessary. Therefore, an amyloid formulation may allow sustained release of the peptide drug for a long duration, thus avoiding the need of repetitive dosing, surgery for implanting depots, or/and complicated manufacturing procedures for making a depot filled with the drug.

To test the concept that amyloid properties of peptides/proteins may be used for the formulation of long-acting drugs, we studied GnRH analogs because long-acting GnRH analogs form gels [29], which is one of the indicators for fibril formation. Indeed, in vitro analysis of a family of 23 short- and long-acting analogs shows that all of the long-acting analogs studied form fibrils that slowly release the peptide monomer (Table 1). Furthermore, we demonstrated that the long-acting analogs also have the capacity to form amyloids in vivo upon subcutaneous injection (Figure 4). In contrast to long-acting analogs, the short-acting GnRH analogs either do not form fibrils, form unstable fibrils, which release peptide quickly, or form very stable fibrils, which do not release peptide within the period studied (Table 1). In addition, we show that the administration of a fibrillar sample results in a longer duration of action than its corresponding soluble counterpart (Figure 5). The findings strongly suggest that the long duration of action is directly related to the ability of the GnRH analog to form fibrils that release sustained peptide (Figure 8). The in vitro half-life time of amyloids of GnRH analogs varies between 2 d to more than 35 d, the *K*_off_ varies more than one order of magnitude as well (Table 1). This observation also shows that the release of monomer from the amyloid depot can be controlled by the chemical structure of the GnRH analog. Concomitantly, the duration of action can be manipulated through the peptide release property of the amyloid fibril by the chemical structure of the analog.

Since amyloid fibrils of disease-specific peptides/proteins have been associated with a number of diseases, a potential pitfall of the use of amyloid as storage depot could be amyloid-associated toxicity of the depot as well as its potential seeding capacity to amplify amyloids by recruiting soluble host counterpart, i.e., wild-type GnRH in the context of this study. Furthermore, the depot might cross-seed other amyloidogenic proteins. The in vitro seeding experiments suggested that amyloid of the GnRH analog L20 can seed neither wild-type GnRH nor α-Syn, a peptide associated with Parkinson disease (Figures S7 and 7). The rationale of this lack of seeding is based on the finding that seeding requires a host counterpart which has the ability to form fibrils and comprises a high sequence homology to the amyloid [1,45]. Since, GnRH does not form fibrils (Table 1, S12) and α-Syn has no significant sequence homology with L20, seeding is unlikely. In addition, the potential capacity of cross-seeding of the depot in vivo is also unlikely due to the different compartmental localization of the seed and its host counterpart, i.e., amyloids of GnRH analogs in the subcutaneous layer and α-Syn in the brain. The brain and the subcutaneous layer are additionally separated by the blood–brain barrier, inhibiting the crossing of peptides to the central nervous system (CNS). Furthermore, amyloids are not toxic to dermal cells (Figure 6), and long-acting analogs show good properties in histamine release [43].

One of the long-acting, amyloid-forming GnRH analogs under study is degarelix (L13). Its pharmacological profile has been well studied. Degarelix (L13) has a good safety margin with regard to histamine release and long duration of action up to 50 d [30] attributed here to the formation of amyloids. Degarelix (L13) is in clinical trials for the treatment of prostate cancer. The results of the Phase IIb clinical trial show that treatment with degarelix (L13) results in fast, profound, and sustained reductions in testosterone and prostate-specific antigen levels without a testosterone surge. Degarelix (L13) is currently being tested in Phase III trials [57]. Another long-acting and amyloid-forming GnRH analog studied here is ganirelix (L23), which is an approved drug (brand name, Antagon) for use in assisted reproduction to control ovulation. Our data suggest that its long duration of action is also because of its ability to form amyloid fibrils.

Since the formation of amyloid is probably a generic feature of the polypeptides, the concept of designing peptide/protein drugs that make amyloid depots for the formulation of long-acting drugs could be applicable to many drugs of peptide or protein origin. Primary requirements of this approach are that monomers that escape from the fibril end be functional and that a given designed segment, which may include, if necessary, an amyloidogenic tag, of the peptide/protein drug forms an amyloid core. The successful application to treat human diseases using long-acting, amyloid forming GnRH analogs such as degarelix and ganirelix underline the potential of the concept. It follows that in striking contrast to the original association of amyloids with diseases, amyloids might also be useful in the treatment of diseases.

## Materials and Methods

### Peptide synthesis.

Peptides were synthesized using the solid-phase approach and the Boc strategy as reported earlier [29].

### Peptide fibril formation.

GnRH analogs were dissolved in a glass tube in 1 ml of 5% D-mannitol and 0.01% sodium azide at a concentration of 1 mg/ml. The GnRH analogs were then incubated at room temperature without stirring. The fibril formation was monitored by EM, CR, and time-resolved by Thio T binding studies. Three independent experiments were performed for each sample.

### Thio T binding.

A 10-μl aliquot of peptide sample was diluted into 500 μl of water with 5% D-mannitol containing 0.01% (w/v) sodium azide. The solution was mixed with 10 μl of 1 mM Thio T prepared in the same solution. Fluorescence was measured immediately after addition of Thio T. The experiment was measured on a spectrofluorimeter (Photon Technology International) with excitation at 450 nm and emission at 482 nm. The fluorescence intensity at 482 nm was plotted against incubation time (day). A rectangular 10-mm quartz microcuvette was used. Three independent experiments were performed for each sample.

### CR binding.

A 5-μl aliquot of peptide sample was mixed with 80 μl of PBS buffer containing 10% ethanol. Then 15 μl of a 100 μM CR solution (filtered through 0.2-μm filter) in PBS containing 10% ethanol was added. After mixing, UV was measured from 300–700 nm. For the measurement of the CR-only spectrum, 15 μl of CR solution with 85 μl of PBS containing 10% ethanol was prepared. As a control, a 5-μl aliquot of GnRH analog mixed with 95 μl of PBS containing 10% ethanol was measured. Three independent experiments were performed for each sample. In addition to the UV measurements, pictures of the cuvette containing the peptide sample and CR were taken to visually show the CR binding to the amyloids.

### Electron microscopy.

A 5-μl aliquot of peptide sample was diluted into 50 μl of water to a peptide concentration of approximately 50 μM, spotted on a glow-discharged, carbon-coated Formvar grid (Electron Microscopy Sciences), incubated for 5 min, washed with distilled water, and then stained with 1% (w/v) aqueous uranyl formate solution. Uranyl formate solutions were filtered through 0.2-μm sterile syringe filters (Corning) before use. EM analysis was performed using a JEOL JEM-100CXII electron microscope at 80 kV with nominal magnifications between 36,000 and 72,000. Images were recorded digitally by using the SIS Megaview III imaging system. At least two independent experiments were carried out for each sample.

### Effect of LMW heparin on amyloid formation.

The 600 μM L13 and L14 hormone analogs were mixed with 600 μM LMW heparin (5-kDa heparin from CalBioChem) in 5% D-mannitol, 0.01% sodium azide (1:1 v/v). The mixtures were incubated overnight. For the control, 600 μM L13 and L14 hormone analogs were also incubated with 5% D-mannitol (1:1 v/v). The next day, a 10-μl aliquot of peptide sample was diluted into 500 μl of H_2_O with 5% D-mannitol containing 0.01% (w/v) sodium azide. The solution was mixed with 10 μl of 1 mM Thio T prepared in the same solution. Fluorescence was measured immediately after addition of Thio T with a spectrofluorimeter (Photon Technology International) using an excitation wavelength at 450 nm and emission at 482 nm. The fluorescence intensity at 482 nm was plotted. Three independent experiments were performed for each sample.

### Peptide release from amyloid.

A 200-μl aliquot of aged GnRH in solution conditions given above was transferred into a 0.6-ml Eppendorf tube with a hole in the cap. To study the effect of heparin on peptide release, a mixture of hormone analog and heparin (1:1 v/v) was incubated overnight in parallel with a half-diluted peptide fibril sample in 5% D-mannitol and 0.01% sodium azide. The hole in the cap of the Eppendorf tube was sealed with a 3.5-kDa molecular weight cutoff (MWCO) membrane. Next, the Eppendorf tube was inserted upside down into a 15-ml Falcon tube containing 4 ml of 5% D-mannitol in water and 0.01% sodium azide. Care was taken to make sure that the membrane was exposed to solutions both in and outside and that any bubbles at the membrane interface were removed. The tubes were then incubated at room temperature. To measure the remaining peptide concentration inside the 0.6-ml Eppendorf tube, a 5-μl aliquot was diluted into 500 μl water with 5% D-mannitol, 0.01% sodium azide, and the fluorescence was measured with excitation at 280 nm and emission in the range of 290–500 nm. The excitation slit width was 2 nm, and emission slit width was 11 nm. To determine the peptide concentration outside the membrane in the 15-ml tube, an aliquot of 250 μl of solution was taken from the releasing medium, diluted into 500 μl final volume by 5% D-mannitol, 0.01% sodium azide, and fluorescence was measured. Following spectra recording, the dialysis system was reassembled as described above. No significant change (±5 nm) of the wavelength maxima (λ_max_) of the fluorescence spectra from solutions inside and outside of the membrane was observed. Furthermore, an independent experiment, in which the intrinsic fluorescence during aggregation of GnRH analogs was measured over a period of 30 d, did not show significant changes of fluorescence intensity and wavelength maxima from freshly prepared solution to aggregates by 30 d of incubation (unpublished data). The normalized intensity maxima from inside and outside the membrane were plotted as a function of time and fit by an exponential function to extract the half-life of the fibrils and the *K*_off_, respectively. The data from day 0 and day 1 of incubation were not used in the analysis, because loosely bound monomer or monomers that are already in equilibrium with fibrils pass through the membrane during this initial incubation period, resulting in a bi-exponential function, which is difficult to fit. The extracted half-life, *t*_1/2_, is the time taken, during which the fluorescence signal inside the 0.6-ml Eppendorf tube drops by 50%. The dissociation constants (*K*_d_) of the aggregates given are equal to the final monomer concentration in accordance to O'Nuallain et al. [58].

### In vivo rat experiments.

Skin harvest: Adult male Sprague-Dawley rats (180–220 g) were injected subcutaneously in shaved areas with the vehicle, freshly prepared (i.e., L14, S2, S4, and vehicle-only; 200 μg/200 μl at four sites/rat), or aged GnRH analogs (i.e., S2 and S4; 200 μg/200 μl at one site/rat). Twenty-four hours or 7 d later, the animals were deeply anesthetized and tissues, including skin and muscle around the injection sites, were collected. They were then fixed in fresh 4% paraformaldehyde for 7 d, postfixed in 20% sucrose/4% PFA overnight, and divided into three sections per treatment. Each section was cut on cryostat at 10-μm thickness and stained with CR.

*LH levels*: Adult male Sprague-Dawley rats (180–200 g were castrated under isoflurane anesthesia 10 d prior to the start of the experiment, and implanted with indwelling jugular cannulae 48 h prior to the assay for serial blood sampling [59]. The peptides (200 μg) were dissolved either fresh in 200 μl of bacteriostatic water containing 5% mannitol or aged under the same conditions for 4 mo. The rats were injected subcutaneous with a total dose of 50 μl. Controls received the vehicle. Plasma LH levels were determined by radioimmunoassay (RIA) using reagents provided by the National Pituitary and Hormone Distribution Program of the National Institute of Diabetes and Digestive and Kidney Diseases (NIDDK; Bethesda, Maryland) [60]. All protocols were approved by the Salk Institute's Institutional Animal Care and Use Committee (IACUC).

### In-cell CR birefringence.

The CR staining was performed using the diagnostic amyloidal stain kit HT60 from Sigma. Briefly, microscopic slides containing tissue were stained with hematoxylin solution (Gill No. 3) for 10 min and rinsed in tap water for 5 min. Slides were placed in alkaline sodium chloride solution for 20 min and subsequently stained in alkaline CR solution for 20 min. The slides were rinsed three times with absolute ethanol and cleared in xylene before mounting. The samples were analyzed using a microscope equipped with a dual polarizer and a charge-coupled device (CCD) camera.

### LDH release assay.

For the LDH assay, we used mouse embryo fibroblast cells and peptide fibrils at 60 μM final concentration in 5% D-mannitol. For the buffer control, we used 5% D-mannitol in water. We also used 1 μM Aβ(1–40) and a cell lysate to compare our data. The assay was done using a kit (Sigma) as described by the manufacturer. The assays were done in triplicate and the data presented as the standard error of the mean.

### Seeding experiment.

A total of 5 μl of matured L20 fibril solution at a concentration of 1 mg/ml in 5% D-mannitol, 0.01% sodium azide was mixed with 95 μl of freshly prepared GnRH solution in the same solution condition followed by incubation at room temperature without stirring. At day 0 and day 30, aliquots of the mixture as well as the original solution were taken for EM. Once a week, a 10-μl aliquot was diluted into 500 μl water containing 5% D-mannitol, 0.01% sodium azide, and 10 μl of 1 mM Thio T to measure the Thio T fluorescence as described above. Wild-type α-Syn was expressed and purified according to Kessler et al. [47]. Solid σ-Syn was dissolved in PBS (pH 7.4), 0.01% sodium azide at a concentration of 10 mg/ml. The pH was adjusted to 7.4 by adding the appropriate amount of NaOH solution. The solution was briefly vortexed and then transferred to filters (50,000 MWCO, Microcon YM-50; Millipore), and centrifuged for 30 min at 16,000*g* using a benchtop microcentrifuge (Eppendorf model 5417R; Brinkmann Instruments). The cutoff filters were washed three times by centrifuging with 200 μl of PBS (pH 7.4) before use. The resulting solution should contain only monomer or dimer wild-type α-Syn. The protein concentration of the supernatant was measured by UV absorbance with ɛ_280_ of 6,500 M^−1^cm^−1^. The 400 μM α-Syn solution was incubated with 5% (v/v) seed of matured L20 amyloid in 5% D-mannitol at 37 °C with vigorous stirring. α-Syn with only 5% D-mannitol was also incubated as a control. The monomeric solution of α-Syn was incubated with 5% (v/v) of sonicated α-Syn fibril seed as a positive seeding control. The formation of amyloid was monitored regularly for the mixture (α-Syn+L20) and α-Syn by Thio T binding.

## Supporting Information

Figure S1 CR Binding of GnRH Analogs Incubated for 30 d The GnRH analogs are labeled in accordance to Tables 1, S1, and S2. Pictures of a 10-mm fluorescence cuvette containing the peptide sample upon CR binding were taken to show visually the CR binding to the amyloids. “C” indicates control of only CR solution.
(285 KB PDF)

Figure S2 Morphologies of GnRH Analogs Incubated for 30 d Transmission electron microscopy (TEM) was performed on negative-stained samples. The GnRH analogs are labeled in accordance to Tables 1, S1, and S2. Scale bars indicate 200 nm.
(284 KB PDF)

Figure S3 In Vitro Release Profiles of GnRH Analogs from Aggregates As a supplement to Figure 3, the fluorescence spectra and the release profiles of S1, S2, S3, and L15 are shown. The fluorescence spectra were measured with excitation at 280 nm and emission at the range of 290–500 nm at day 0, 1, 3, 17, and 35 as indicated. The nomenclature described in Figure 3 is used.
(307 KB PDF)

Figure S4 Thio T Binding of GnRH Analogs in Presence and Absence of LMW Heparin Thio T fluorescence of L13 and L14 after overnight incubation in presence or absence of LMW heparin are presented. The intensity of Thio T fluorescence is shown as arbitrary units (AU).
(41 KB PDF)

Figure S5 Effect of LMW Heparin on Monomer Release Profiles of Long-Acting GnRH Analogs Time-dependent monomer release of aged samples of L14 in absence (control) and in presence of LMW heparin was measured by the intrinsic fluorescence of the GnRH analogs with excitation at 280 nm and emission at the range of 290–500 nm. The volume and concentration corrected fluorescence maxima (*I*_max_) from both inside (in) and outside (out) of the membrane medium were plotted against time of incubation. The same terminology as in Figure 3 is used. The labels “Hep-in” and “Hep-out” are the corresponding labels of the experiment in presence of LMW heparin. The fluorescence intensity is expressed in arbitrary units (AU).
(27 KB PDF)

Figure S6 In Vivo Amyloid Formation of Short-Acting GnRH Analogs The monomer and fibrils preparations of S2 and S4 (200 μg/200 μl) in 5% D-mannitol, 0.01% sodium azide were injected subcutaneously into 2-mo-old rats, which were sacrificed 1 d and 7 d later, respectively. Tissue sections close to the injection site were stained with CR. The pictures on the left side represent the bright-field microscope images with 10× resolutions. The same sections are shown on the right side under cross-polarized light with 10× magnification.
(A) Tissue section harvested 24 h upon injection with an aged S2 sample that shows amyloid fibrils under the microscope (unpublished data).
(B) Corresponding tissue section upon injection of freshly prepared S4 representing monomeric S4.
(C) Corresponding tissue section upon injection of an aged S4 sample, which shows amyloid fibrils under the microscope.
(D) and (E) Tissue sections harvested after 7 d upon injection of an aliquot of the same fresh and aged S4 samples used in (B) and (C).
(724 KB PDF)

Figure S7 Lack of Seeding Capacity of L20 Fibrils on Wild-Type GnRH (S12) GnRH (S12) was incubated with a seed of matured L20 amyloids (5% v/v) at room temperature for 1 mo in 5% D-mannitol, 0.01% sodium azide, without stirring. The formation of amyloid was monitored for the mixture (L20+S12), matured L20, as well as S12 only by (A) Thio T binding in a time-resolved manner and by (B) EM at day 0 and day 30.
(A) The intensity of Thio T fluorescence is shown as arbitrary units (AU) versus time.
(B) Scale bars indicate 200 nm.
(248 KB PDF)

Table S1 Short-Acting GnRH Analogs with Their Duration of Action (85 KB DOC)

Table S2 Long-Acting GnRH Analogs with Their Duration of Action (82 KB PDF)

## Abbreviations

CR: Congo red
EM: electron microscopy
LDH: lactate dehydrogenase
LH: luteinizing hormone
LMW: low molecular weight
Thio T: Thioflavin T
α-Syn: α-synuclein
