# Supplementary material for: Amyloid as a Depot for the Formulation of Long-Acting Drugs
Source: PLoS Biol. 2008 Feb 5;6(2):e17. doi: 10.1371/journal.pbio.0060017 (PMC2225439; doi:10.1371/journal.pbio.0060017)
Supplement: Supplementary file 2 [file pbio.0060017.sg002.pdf]

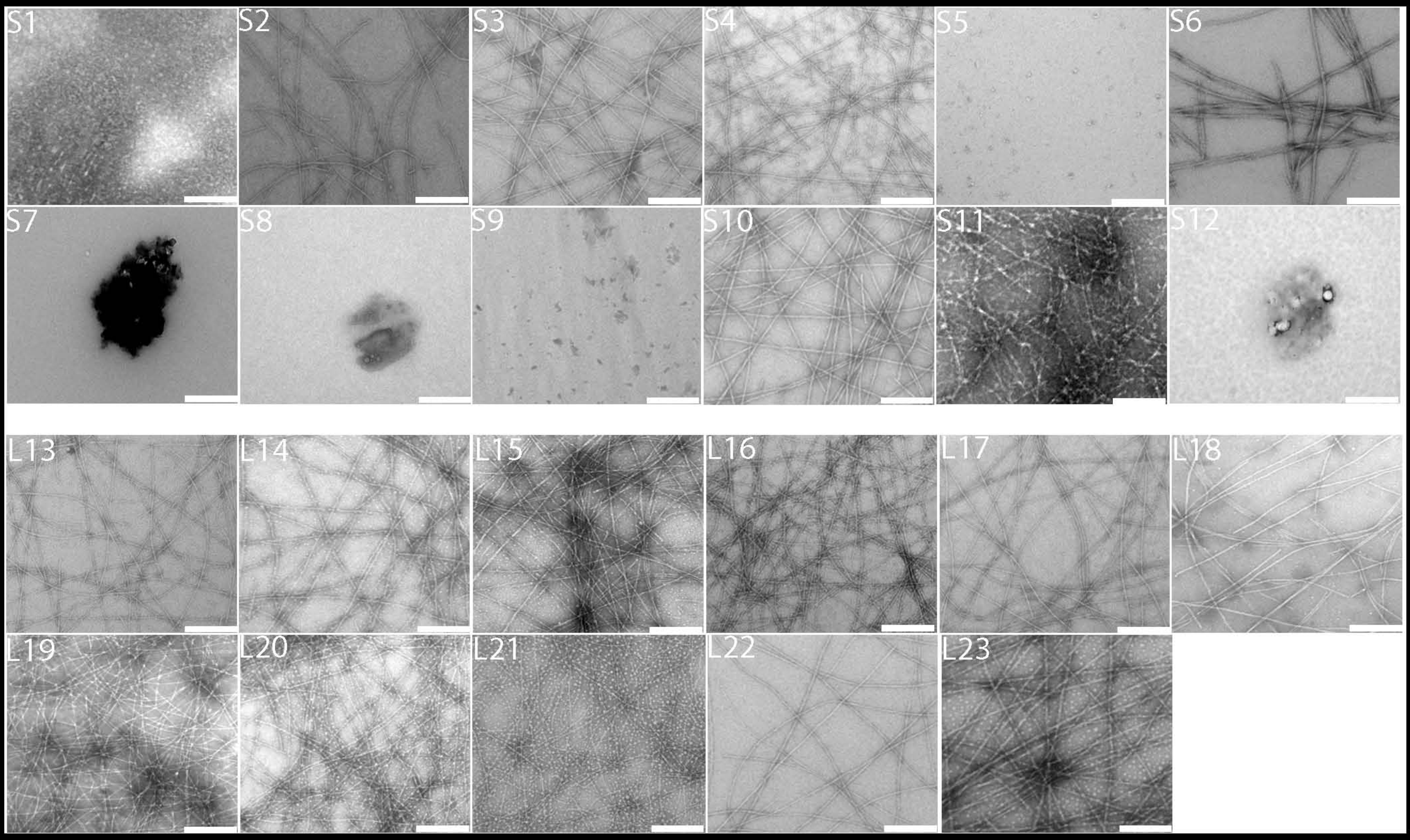
S1
S2
S3
S4
S5
S6
S7
S8
S9
S10
S11
S12
L13
L14
L15
L16
L17
L18
L19
L20
L21
L22
L23
