# Supplementary material for: Amyloid as a Depot for the Formulation of Long-Acting Drugs
Source: PLoS Biol. 2008 Feb 5;6(2):e17. doi: 10.1371/journal.pbio.0060017 (PMC2225439; doi:10.1371/journal.pbio.0060017)
Supplement: Supplementary file 3 [file pbio.0060017.sg003.pdf]

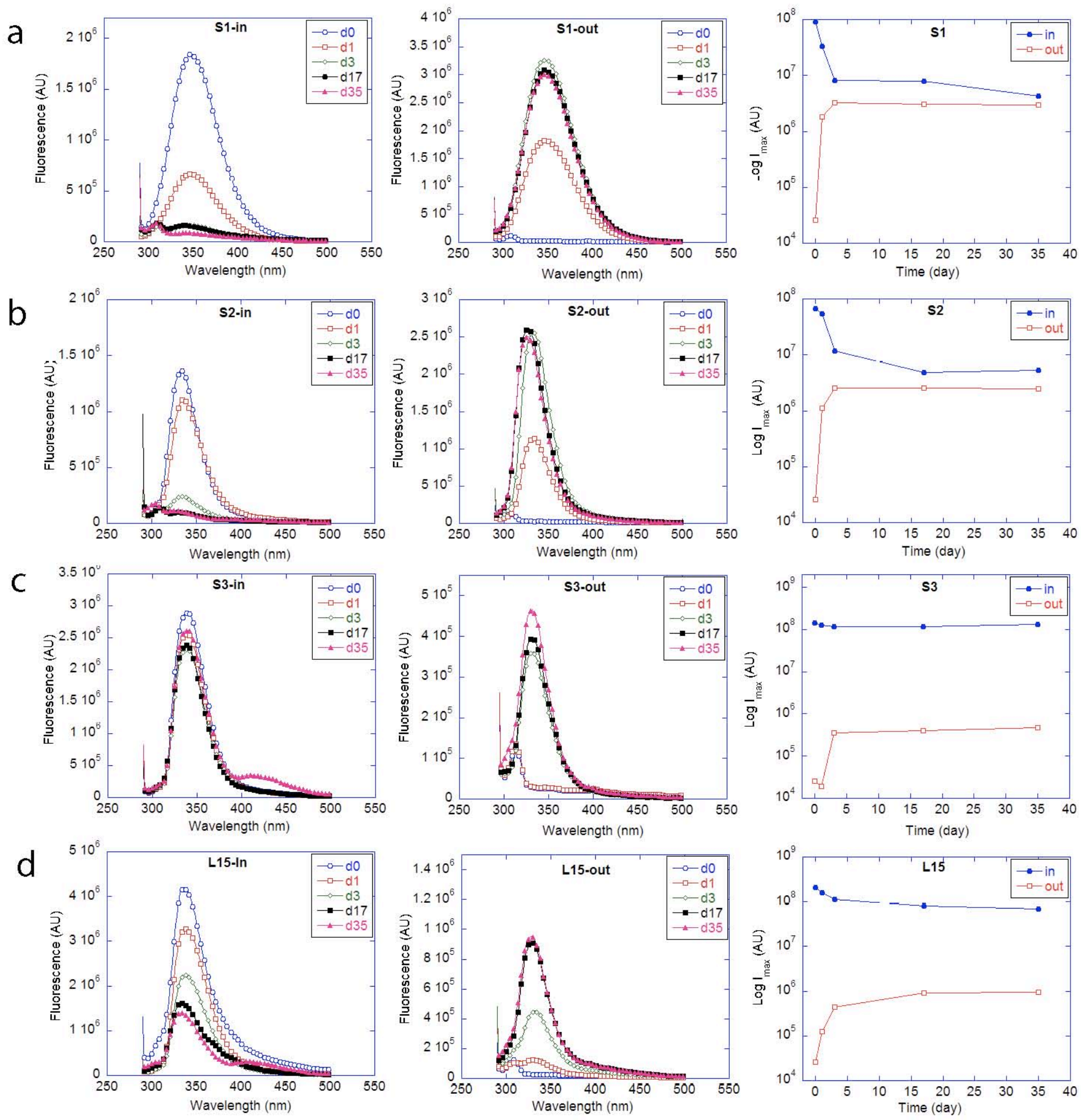
a
b
c
d
S1-in
S1-out
S1
S2-in
S2-out
S2
S3-in
S3-out
S3
L15-in
L15-out
L15
d0
d1
d3
d17
d35
in
out
Fluorescence (AU)
Wavelength (nm)
Log I max (AU)
Time (day)
