# Supplementary material for: Amyloid as a Depot for the Formulation of Long-Acting Drugs
Source: PLoS Biol. 2008 Feb 5;6(2):e17. doi: 10.1371/journal.pbio.0060017 (PMC2225439; doi:10.1371/journal.pbio.0060017)
Supplement: Supplementary file 4 [file pbio.0060017.sg004.pdf]

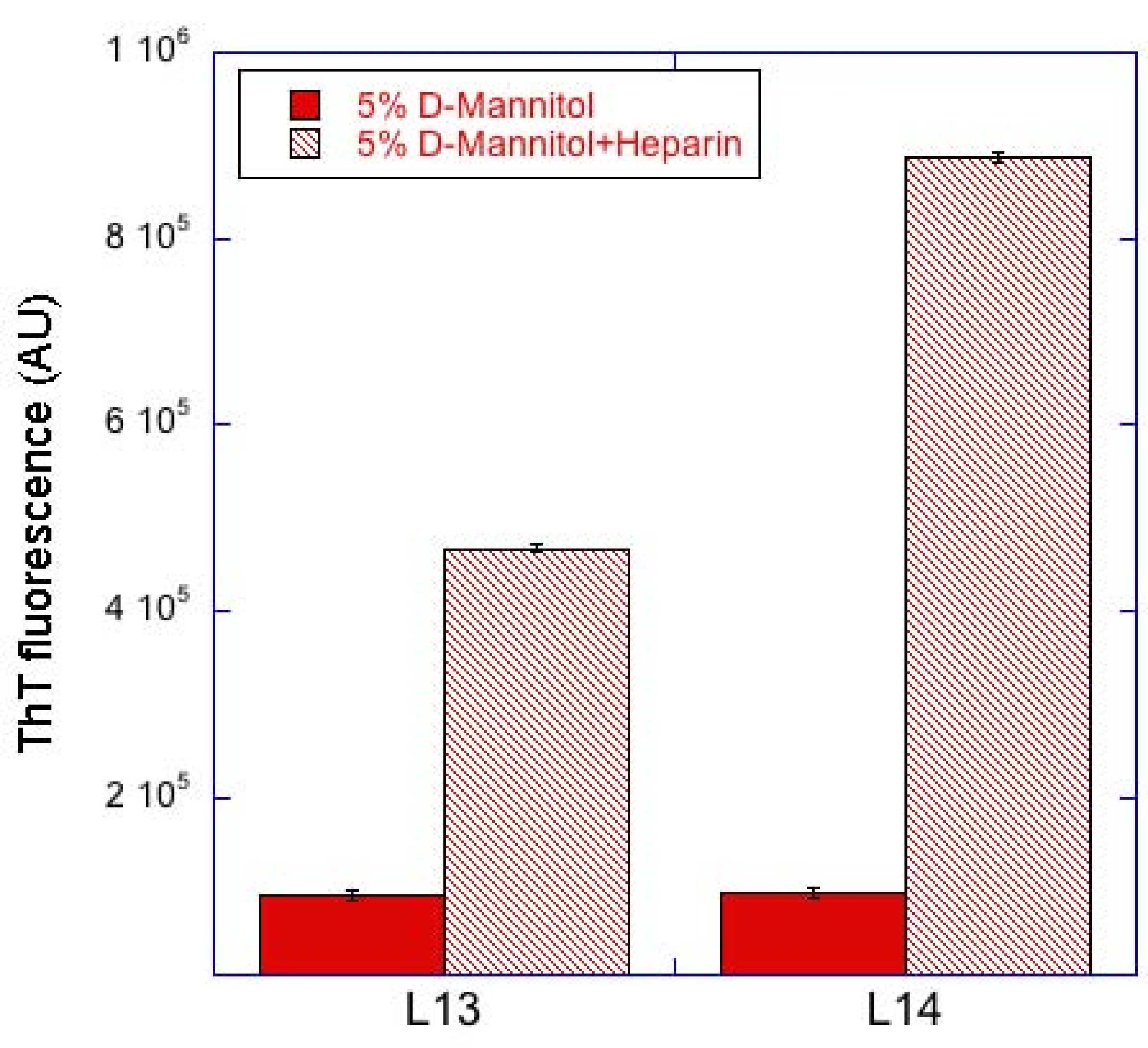

5% D-Mannitol
5% D-Mannitol+Heparin
ThT fluorescence (AU)
1 10^6
8 10^5
6 10^5
4 10^5
2 10^5
L13
L14
