# Supplementary material for: Amyloid as a Depot for the Formulation of Long-Acting Drugs
Source: PLoS Biol. 2008 Feb 5;6(2):e17. doi: 10.1371/journal.pbio.0060017 (PMC2225439; doi:10.1371/journal.pbio.0060017)
Supplement: Supplementary file 5 [file pbio.0060017.sg005.pdf]

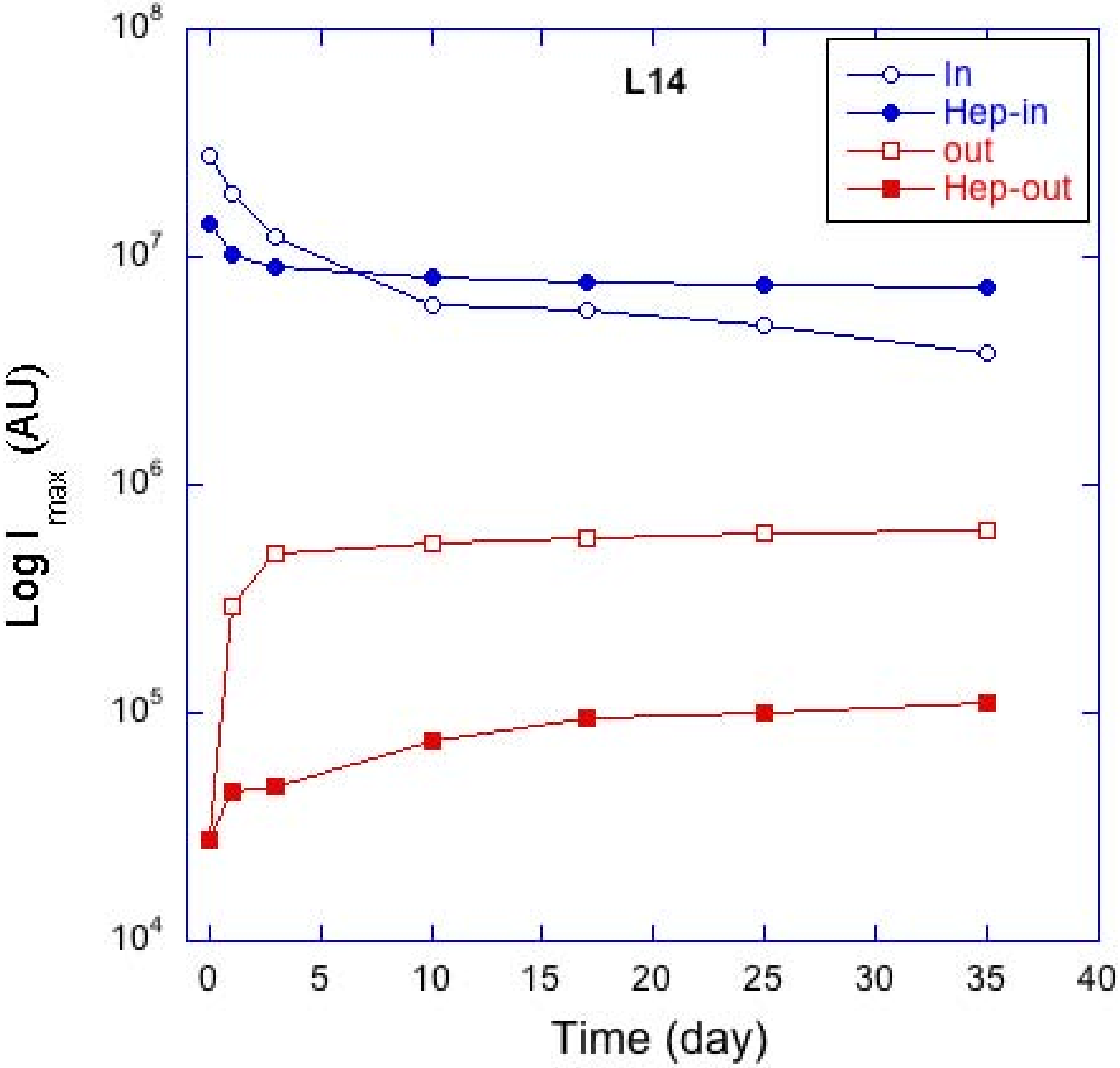
L14
In
Hep-in
out
Hep-out
$10^8$
$10^7$
$10^6$
$10^5$
$10^4$
Log $I_{max}$ (AU)
0
5
10
15
20
25
30
35
40
Time (day)
