# Supplementary material for: Amyloid as a Depot for the Formulation of Long-Acting Drugs
Source: PLoS Biol. 2008 Feb 5;6(2):e17. doi: 10.1371/journal.pbio.0060017 (PMC2225439; doi:10.1371/journal.pbio.0060017)
Supplement: Supplementary file 7 [file pbio.0060017.sg007.pdf]

a

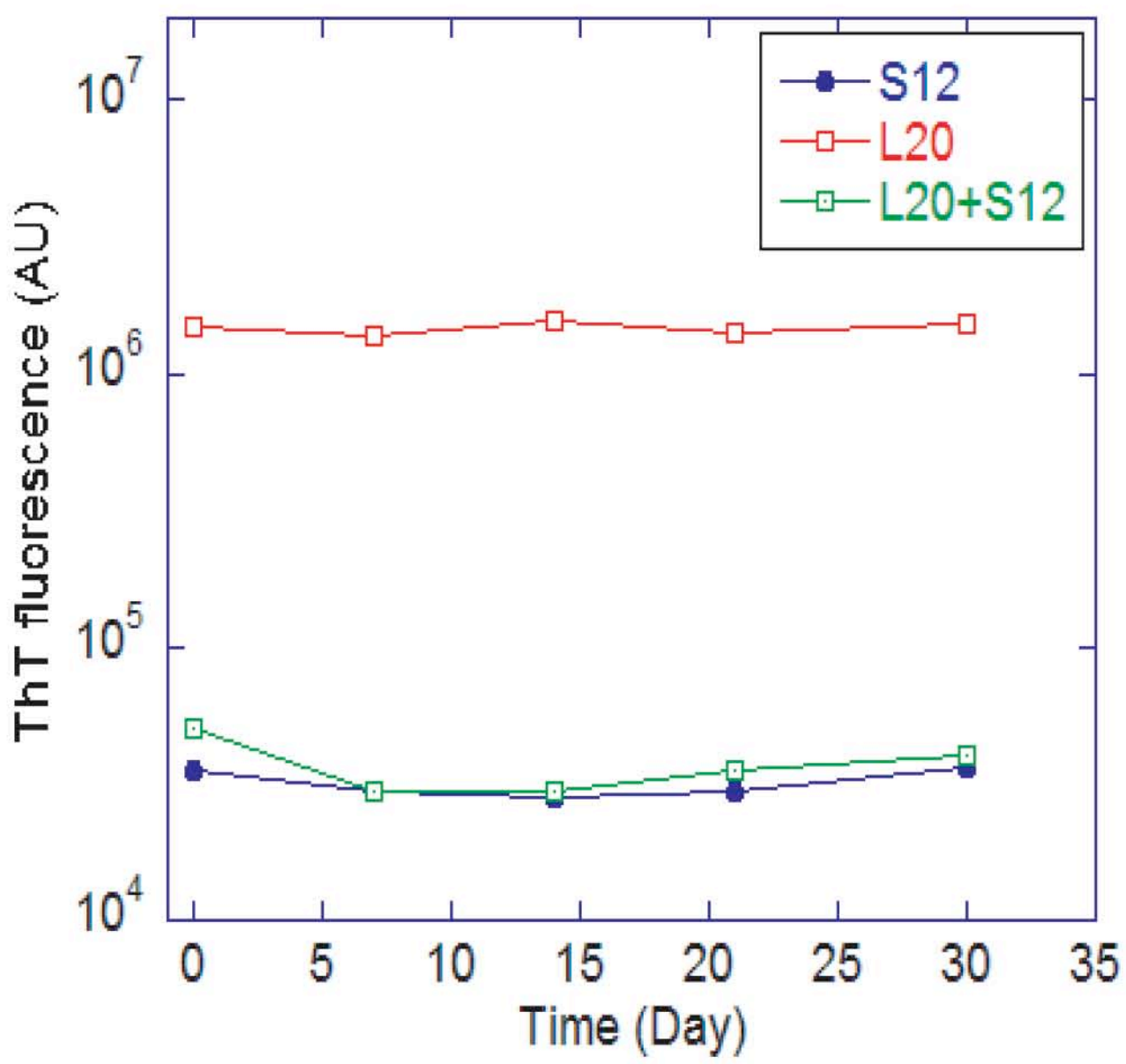
S12
L20
L20+S12
10^7
10^6
10^5
10^4
ThT fluorescence (AU)
0
5
10
15
20
25
30
35
Time (Day)

b

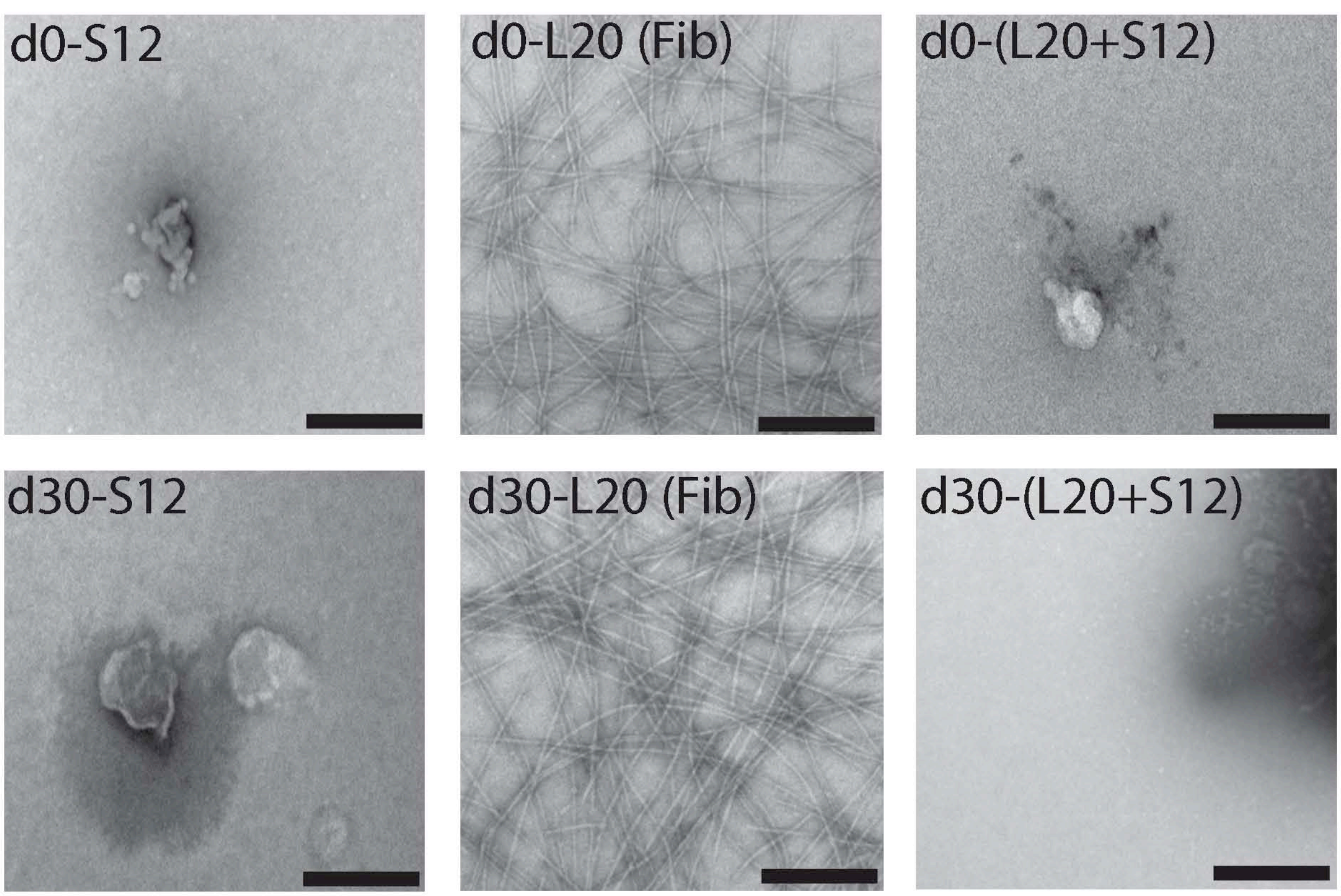
d0-S12
d0-L20 (Fib)
d0-(L20+S12)
d30-S12
d30-L20 (Fib)
d30-(L20+S12)
