# Supplementary material for: Amyloid as a Depot for the Formulation of Long-Acting Drugs
Source: PLoS Biol. 2008 Feb 5;6(2):e17. doi: 10.1371/journal.pbio.0060017 (PMC2225439; doi:10.1371/journal.pbio.0060017)
Supplement: Supplementary file 8 [file pbio.0060017.st001.doc]

**Table S1: Short acting GnRH analogs with their duration of action**

| No | Compound | IC_50_^a^  (nM) | Duration of action^b^ | References |
| --- | --- | --- | --- | --- |
| S1 | [D(Im-bzl)His^6^,Pro^9^-NHEt]GnRH (histrelin, Agonist) |  | Short | [1] |
| S2 | [Aph(CO-(C_2_H_4_O)_3_-C_2_H_4_-NH-CO-NH-OCH_3_)^5^]degarelix | 1.06 | Short | [2] |
| S3 | des-Ac-[D-Aph(CO-NH-OCH_3_)^6^]-C_2_H_5_-(C_2_H_4_O)_2_-NH-CO-degarelix | 1.61 | Short | [2] |
| S4 | des-Ac-[Aph(CO-NH-OCH_3_)^5^,D-Aph(CO-NH-OCH_3_)^6^]-CH_3_-(CH_2_)_2_-CO-degarelix | 1.37 | Short | [2] |
| S5 | Ac-D3-Pro-DFpa-DTrp-Ser-Tyr-DTrp-Leu-Arg-Pro-Gly-NH_2_ (4F antagonist) | 0.13 | Short | [3] |
| S6 | Ac-DNal-DCpa-DPal-Ser-Lys(Nic)-DLys(Nic)-Leu-ILys-Pro-DAla-NH_2_ (Antide) | 4.4 | Short | [4] |
| S7 | Ac-DNal-DCpa-DPal-Ser-Arg-DGlu(AA)-Leu-Arg-Pro-DAla-NH_2_ (Nal-Glu antagonist) | 3.3 | Short | [5] |
| S8 | Ac-Agl(Me,2-Napht)-DCpa-DPal-Ser-Aph(Ac)-DAph(Ac)-Leu-ILys-Pro-DAla-NH_2_ | 0.30 | V. short | [6] |
| S9 | Ac-DNal-DCpa-DPal-Ser-D,LAgl(Me,acetylamino)benzoyl-DAph(Ac)-Leu-ILys-Pro-DAla-NH_2_ | 0.43 | V. short | [6] |
| S10 | des-Ac-[DAph(CONHOCH3)6]Peg-degarelix | 9.47 | Short | [2] |
| S11 | [DAph(CONH(C2H4O)2-C2H5]degarelix | 0.88 | Short | [2] |
| S12 | pyro-Glu-His-Trp-Ser-Tyr-Gly-Leu-Arg-Pro-Gly-NH_2_ (GnRH) |  | Short | [7] |

**^a^**IC_50_ is the concentration of antagonist required to repress the GnRH induced response by 50% in the reporter gene assay in HEK-293 cells expressing the human GnRH receptor and a GnRH-responsive stably integrated luciferase reporter gene. The experiments have been measured with fresh soluble material.

^b^Castrated male rat assay. Duration of action: very long = over 80% inhibition of LH (luteinizing hormone) release for more than 120 h; long* = over 80% inhibition of LH release at 96 h but not at 120 h; long = over 80% inhibition of LH release at 72 h but not at 96 h; short = over 80% inhibition of LH release at 3 h but not at 72 h.

**References:**

1.	Doelle GC, Evans RM, Alexander AN, Rivier J, Vale W, et al. (1986) Single dose responses to the gonadotropin-releasing hormone agonist analogue [(imBz 1)-D-His6,Pro9-NEt]GnRH. Horm Metab Res 18:201-202.

2.	Samant MP, Hong DJ, Croston G, Rivier C, Rivier J (2006) Novel analogues of degarelix incorporating hydroxy-, methoxy-, and pegylated-urea moieties at positions 3, 5, 6 and the N-terminus. Part III. J Med Chem 49:3536-3543.

3.	Rivier JR, C.; Perrin, M.; Porter, J.; Vale, W. (1981) GnRH analogs Structure-activity relationships. LHRH Peptides as Female and Male Contraceptives (Harper and Row, Philadelphia, PA).

4.	Ljungqvist A, Feng DM, Hook W, Shen ZX, Bowers C, et al. (1988) Antide and related antagonists of luteinizing hormone release with long action and oral activity. Proc Natl Acad Sci U S A 85:8236-8240.

5.	Jiang G, Stalewski J, Galyean R, Dykert J, Schteingart C, et al. (2001) GnRH antagonists: a new generation of long acting analogues incorporating p-ureido-phenylalanines at positions 5 and 6. J Med Chem 44:453-467.

6.	Jiang G, Miller C, Koerber SC, Porter J, Craig AG, et al. (1997) Betidamino acid scan of the GnRH antagonist acyline. J Med Chem 40:3739-3748.

7.	Matsuo H, Baba Y, Nair RM, Arimura A, Schally AV (1971) Structure of the porcine LH- and FSH-releasing hormone. I. The proposed amino acid sequence. Biochem Biophys Res Commun 43:1334-1339.
