# Supplementary material for: Amyloid as a Depot for the Formulation of Long-Acting Drugs
Source: PLoS Biol. 2008 Feb 5;6(2):e17. doi: 10.1371/journal.pbio.0060017 (PMC2225439; doi:10.1371/journal.pbio.0060017)
Supplement: Supplementary file 9 [file pbio.0060017.st002.doc]

**Table S2: Long acting GnRH analogs with their duration of action**

| No | Compound | IC_50_^a^  (nM) | Duration of action^b^ | References |
| --- | --- | --- | --- | --- |
| L13 | Ac-DNal-DCpa-DPal-Ser-Aph(LHor)-DAph(Cbm)-Leu-ILys-Pro-DAla-NH_2_ (degarelix) | 0.58 | V. long | [1] |
| L14 | [Aph(CO-NH-OCH_3_)^5^]degarelix | 0.60 | Long | [1] |
| L15 | [Aph(CO-NH-(C_2_H_4_O)_2_-C_2_H_5_)^5^]degarelix | 3.16 | Long* | [1] |
| L16 | des-Ac-[Aph(CO-NH-OCH_3_)^5^]-CH_3_-(CH_2_)_2_-CO-degarelix | 1.46 | Long* | [1] |
| L17 | [D-Aph(CO-NH-OCH_3_)^6^]degarelix | 0.51 | Long* | [1] |
| L18 | Ac-DNal-DCpa-DPal-Ser-Aph(Ac)-DAph(Ac)-Leu-ILys-Pro-DAla-NH_2_ (Acyline) | 0.69 | Long | [1] |
| L19 | Ac-DNal-DCpa-DPal-Ser-Aph(Atz)-DAph(Atz)-Leu-ILys-Pro-DAla-NH_2_ (Azaline B) | 1.36 | Long | [1] |
| L20 | Ac-DNal-DCpa-Agl(Nic)-Ser-Aph(Ac)-DAph(Ac)-Leu-ILys-Pro-DAla-NH_2_ | 0.63 | Intermediate | [2] |
| L21 | Ac-Agl(2-Napht)-DCpa-DPal-Ser-Aph(Ac)-DAph(Ac)-Leu-ILys-Pro-DAla-NH2 | 3.0 | Intermediate | [2] |
| L22 | Ac-DNal-DCpa-DPal-Ser-Aph(Ac)-DAph(Ac)-Leu-Agl(Me,IβAla)-Pro-DAla-NH_2_ | 0.50 | Medium | [2] |
| L23 | Ac-DNal-DCpa-DPal-Ser-Tyr-DHArg(Et_2_)-Leu-HArg(Et_2_)-Pro-DAla-NH_2_ (ganirelix) | 3.6 | Intermediate | [3] |

**^a^**IC_50_ is the concentration of antagonist required to repress the GnRH induced response by 50% in the reporter gene assay in HEK-293 cells expressing the human GnRH receptor and a GnRH-responsive stably integrated luciferase reporter gene.

**^b^**Castrated male rat assay. Duration of action: very long = over 80% inhibition of LH (luteinizing hormone) release for more than 120 h; long* = over 80% inhibition of LH release at 96 h but not at 120 h; long = over 80% inhibition of LH release at 72 h but not at 96 h; short = over 80% inhibition of LH release at 3 h but not at 72 h.

**References:**

1.	Samant MP, Hong DJ, Croston G, Rivier C, Rivier J (2006) Novel analogues of degarelix incorporating hydroxy-, methoxy-, and pegylated-urea moieties at positions 3, 5, 6 and the N-terminus. Part III. J Med Chem 49:3536-3543.

2.	Jiang G, Miller C, Koerber SC, Porter J, Craig AG, et al. (1997) Betidamino acid scan of the GnRH antagonist acyline. J Med Chem 40:3739-3748.

3.	Jiang G, Stalewski J, Galyean R, Dykert J, Schteingart C, et al. (2001) GnRH antagonists: a new generation of long acting analogues incorporating p-ureido-phenylalanines at positions 5 and 6. J Med Chem 44:453-467.
